# A Case of Infective Endocarditis Following Bone Marrow Transplantation for Myelodysplastic Syndrome

**DOI:** 10.7759/cureus.73564

**Published:** 2024-11-12

**Authors:** Tomohiro Nakajima, Yutaka Iba, Tsuyoshi Shibata, Yu Iwashiro, Nobuyoshi Kawaharada

**Affiliations:** 1 Cardiovascular Surgery, Sapporo Medical University, Sapporo, JPN

**Keywords:** aortic valve replacement, bone marrow transplantation, infective endocarditis, myelodysplastic syndrome, prednisolone

## Abstract

A 63-year-old man was diagnosed with myelodysplastic syndrome (MDS) at the age of 62 by the hematology department. The patient underwent four cycles of azacitidine (AZA) therapy, followed by successful bone marrow transplantation (BMT). Subsequently, he was hospitalized twice for graft-versus-host disease (GVHD). Prednisolone was initially administered at 60 mg and was gradually tapered to 10 mg/day. Additionally, the patient was prescribed 10 mg/day of a Janus kinase inhibitor. At age 63, approximately one month prior to admission, he began experiencing recurrent upper respiratory symptoms with fevers of around 37°C. He developed a persistent fever of 38°C, accompanied by dyspnea on exertion, and visited the hematology outpatient clinic. Chest radiography revealed prominent pulmonary congestion, leading to the decision to perform echocardiography, which revealed severe aortic valve regurgitation with vegetation attached to the valve. Laboratory findings included a white blood cell count of 13,200/μL and a C-reactive protein (CRP) level of 13.7 mg/dL. Blood cultures revealed the presence of gram-positive cocci.

As the patient’s respiratory condition progressively worsened, emergency aortic valve replacement was planned. Additionally, because of a history of percutaneous coronary intervention (PCI) at another institution, he was referred for a coronary artery bypass graft (CABG) on the right coronary artery to be performed concurrently. Surgery was performed via median sternotomy under cardioplegic arrest. The aortic valve was perforated at the right coronary cusp and was covered with vegetation. The patient underwent aortic valve replacement with a biological valve, and a saphenous vein graft was used for bypass grafting to the posterior descending branch of the right coronary artery. Postoperatively, antibiotic therapy was administered without infection recurrence. The patient was discharged 47 days postoperatively. This case demonstrated the rapid progression of infective endocarditis following BMT, highlighting the need for prompt recognition and management.

## Introduction

Patients who have undergone bone marrow transplantation (BMT) have a significantly increased risk of various infections due to prolonged immunosuppression, particularly when further complicated by graft-versus-host disease (GVHD) or ongoing immunosuppressive therapy [1]. GVHD, a common post-BMT complication, often necessitates the use of high-dose corticosteroids and other immunosuppressants, which further impair immune function and render patients susceptible to severe infections, including opportunistic pathogens. Infective endocarditis (IE) is a rare but life-threatening complication in immunocompromised patients, requiring prompt recognition and aggressive treatment [2].

IE in immunosuppressed individuals presents unique challenges owing to atypical clinical presentations, rapid disease progression, and a heightened risk of complications. These factors often result in poorer outcomes compared to the general population, emphasizing the need for a high index of suspicion in managing such cases [3]. In the present case, a 63-year-old man, who had previously undergone BMT and subsequent GVHD treatment, developed rapidly progressive IE, ultimately requiring emergency aortic valve replacement. This case provides critical insights into the impact of GVHD and intensive immunosuppression on the onset and progression of IE, highlighting the considerations for future clinical management and surveillance strategies for similarly high-risk patients.

## Case presentation

**Figure 1:**
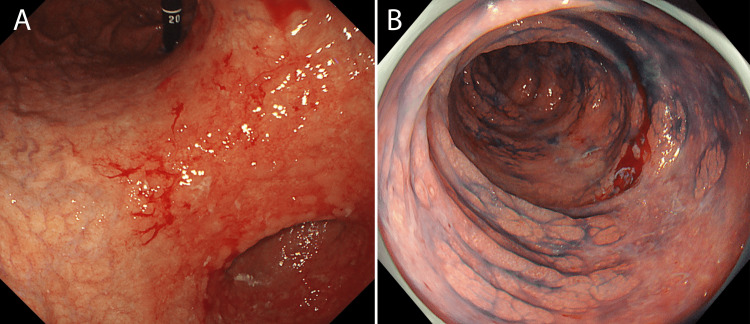
Endoscopic findings (A) Upper gastrointestinal endoscopy: discoloration of the gastric mucosa with areas of minor bleeding observed. (B) Lower gastrointestinal endoscopy: evidence of bleeding sites within the colon.

The patient was a 63-year-old man with a medical history of hypertension and hyperlipidemia, for which he was on medication. At the age of 51, he suffered an acute myocardial infarction, leading to the placement of drug-eluting stents in the circumflex and right coronary arteries. He was followed up regularly thereafter. At 62 years of age, he was diagnosed with myelodysplastic syndrome (MDS) by the hematology department. He completed four cycles of azacitidine (AZA) therapy before undergoing successful BMT. However, he developed GVHD, which required two hospitalizations (Figure 1). His prednisone dose was initially 60 mg, which was gradually tapered to 10 mg/day, and he was taking 10 mg/day of a Janus kinase inhibitor.

**Figure 2:**
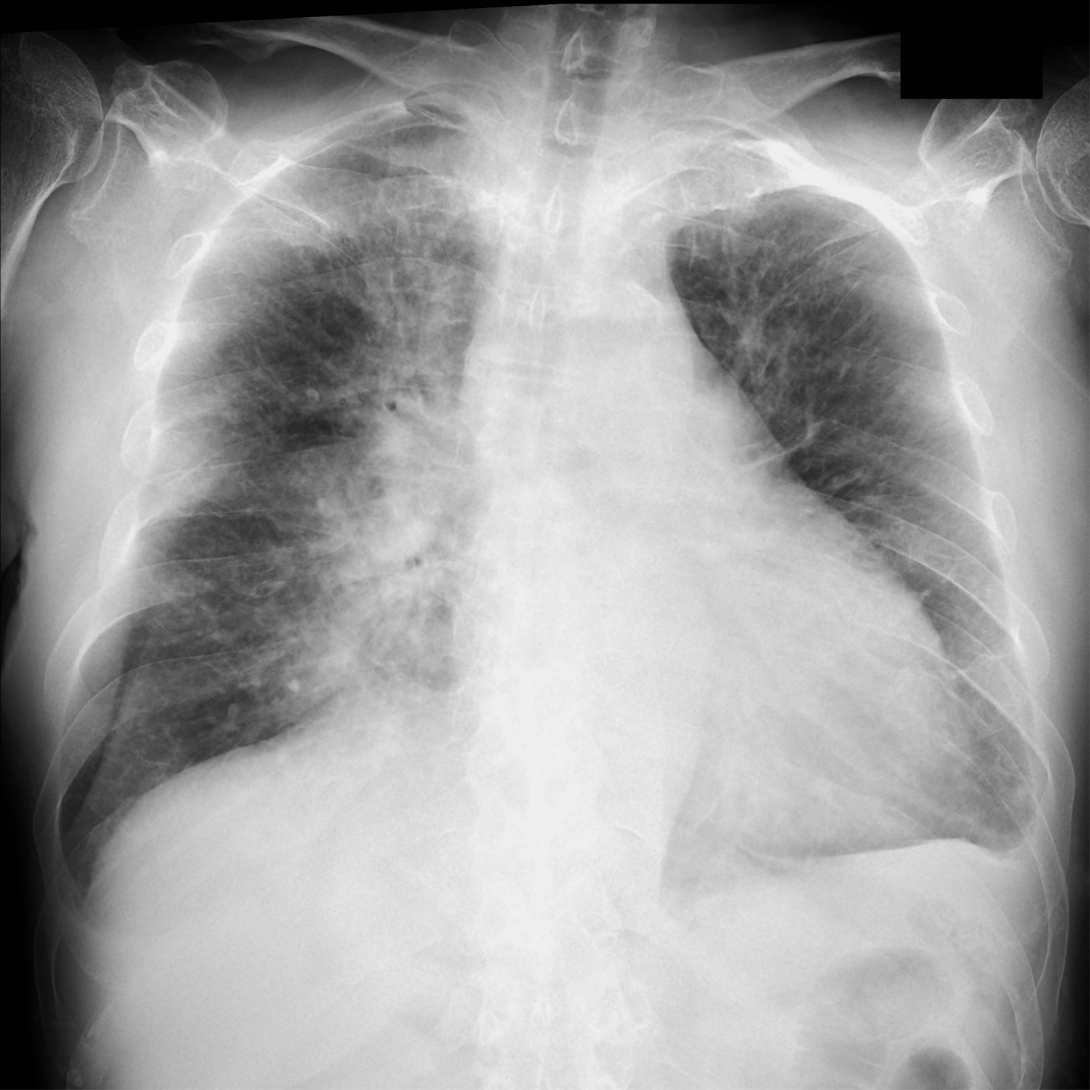
Chest X-ray image Cardiomegaly and marked pulmonary congestion are visible in both lung fields.

**Figure 3:**
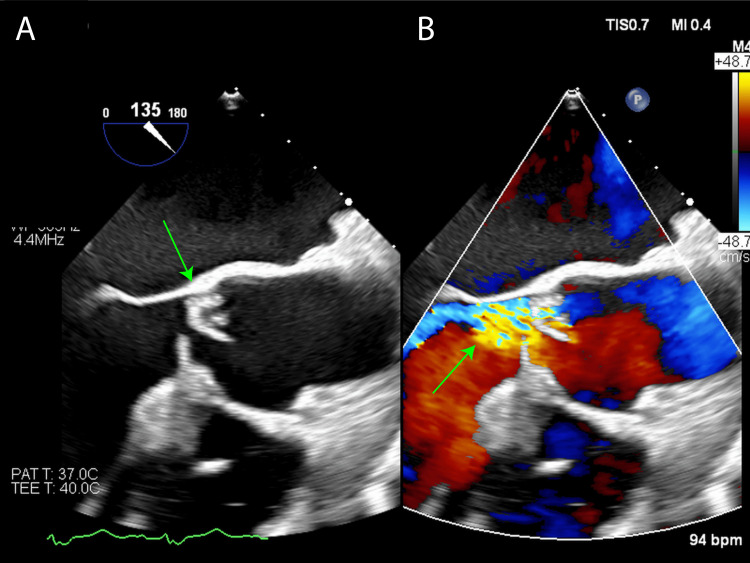
Intraoperative transesophageal echocardiography images (A) Large vegetation attached to the right coronary cusp of the aortic valve (green arrow). (B) Perforation observed on the right coronary cusp of the aortic valve, with a prominent jet of aortic regurgitation (green arrow).

At age 63, approximately one month prior to admission, he began experiencing recurrent upper respiratory symptoms with a low-grade fever of around 37°C. He subsequently developed a persistent fever of 38°C, along with dyspnea upon exertion, prompting a visit to the hematology outpatient clinic. Chest radiography revealed significant pulmonary congestion (Figure 2), leading to an echocardiogram that showed severe aortic regurgitation with vegetative growth on the valve (Figure 3). Laboratory tests indicated a white blood cell count of 13,200/μL and a C-reactive protein (CRP) level of 13.7 mg/dL. Blood cultures revealed gram-positive cocci.

**Figure 4:**
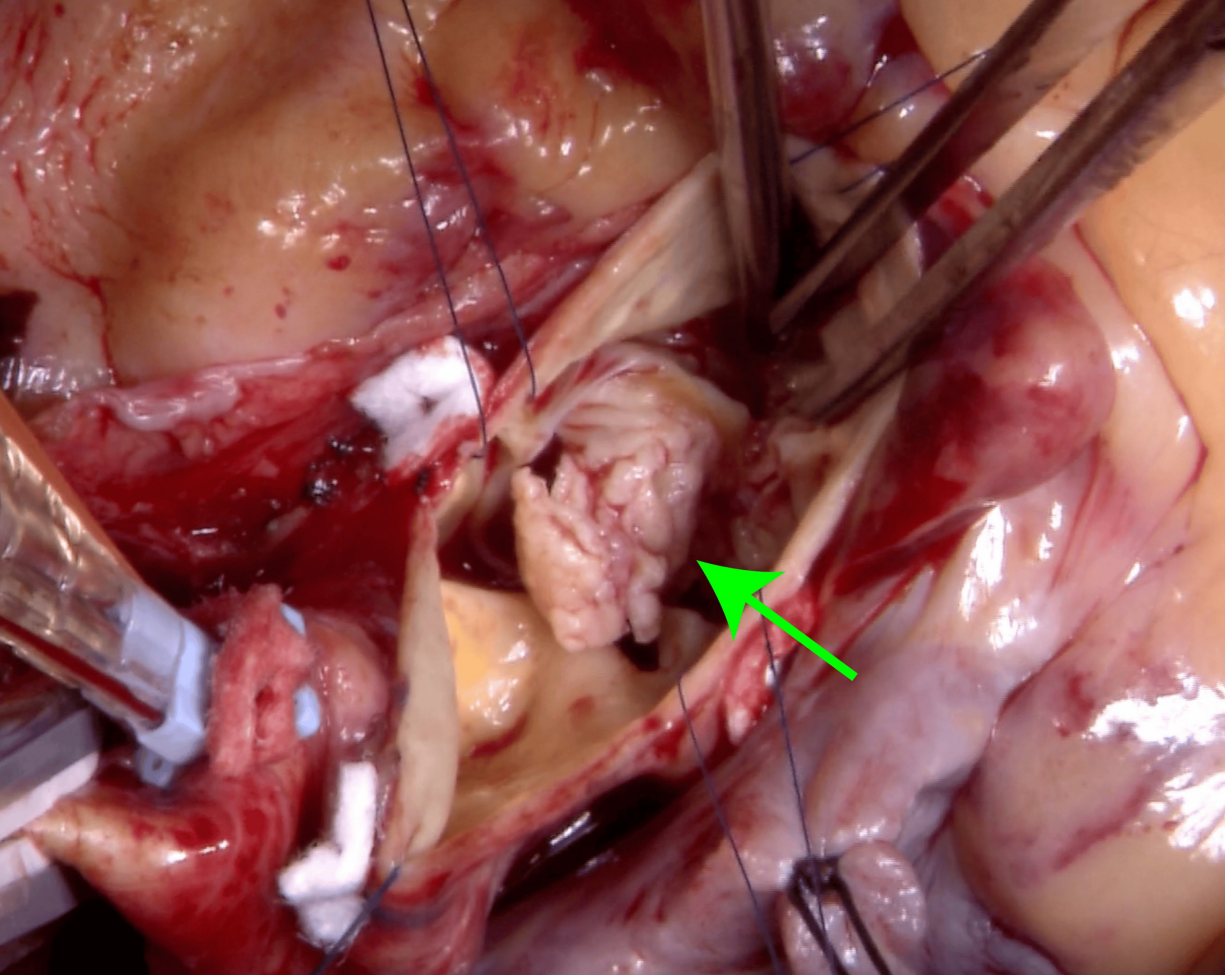
Intraoperative findings Large vegetation attached to the non-coronary cusp of the aortic valve (green arrow).

Due to the worsening respiratory status, emergency aortic valve replacement was scheduled. Additionally, given a history of percutaneous coronary intervention (PCI) at another hospital, a concurrent coronary artery bypass graft (CABG) was recommended for the right coronary artery. Surgery was performed via median sternotomy under cardioplegic arrest. The aortic valve had a perforation at the right coronary cusp with large vegetation (Figure 4). Aortic valve replacement was performed using the INSPIRIS RESILIA aortic bioprosthesis (Edwards Lifesciences LLC, Irvine, USA), placed at 27 mm in the supraannular position. A bypass graft was created in the posterior descending branch of the right coronary artery using the great saphenous vein.

Postoperatively, *Staphylococcus caprae*, which is sensitive to vancomycin, was identified in the blood cultures. The antibiotic regimen was continued, and no reinfections occurred. At the final blood test, the patient’s WBC count was 1900/μL, and CRP was 0.08 mg/dL. The patient was discharged 47 days postoperatively. This case represents the rapid onset of IE following BMT, highlighting the need for prompt diagnosis and intervention.

## Discussion

MDS is a hematological disorder characterized by abnormalities in hematopoietic stem cells, leading to bone marrow cytopenia and dysplasia [4]. MDS primarily affects older adults and can progress to acute myeloid leukemia. Treatment depends on the disease type and severity and may include supportive care, immunosuppressive therapy, low-dose chemotherapy (such as AZA), and hematopoietic stem cell transplantation. Due to immune dysfunction in patients with MDS, there is an elevated risk of infections, including IE, where bacterial colonization of the endocardium is more likely to occur.

Following MDS treatment, patients are often treated with high-dose steroids or immunosuppressants, which induce an immunocompromised state, increasing susceptibility to infections. Although patients are advised to take precautions, infections are sometimes unavoidable. The hematologist instructed her to wear a mask whenever she went out, and she was forbidden to consume any raw food to prevent enteritis. In this case, it is presumed that an upper respiratory infection may have triggered the development of IE [5,6].

Nakajima et al. reported that in cases of IE, it is advisable to attempt a reduction in steroid dosage before surgery if the patient’s condition allows for a delay [7]. With regard to steroids, it is desirable to be able to reduce the dose of prednisolone to 5 mg if possible before surgery. However, in reality, it is not possible to reduce the dose to that level, and the first goal is to reduce the dose to 10 mg as a compromise. However, in the present case, the patient experienced acute aortic regurgitation, which led to rapidly worsening heart failure with hourly clinical deterioration. The presence of large vegetation on the aortic valve, along with the acute onset of aortic insufficiency, made mechanical circulatory support options, such as extracorporeal membrane oxygenation (ECMO), Impella, or intra-aortic balloon pumping, unsuitable [8]. Consequently, emergency surgery was deemed necessary to address the patient’s critical condition.

Through median sternotomy and cardioplegic arrest, aortic valve replacement was performed. The right and left coronary cusps were severely damaged by the infection; however, the infection did not spread to the aortic or mitral annulus, allowing the procedure to conclude with aortic valve replacement alone. Postoperatively, prednisolone and immunosuppressant therapy were continued. To prevent infection recurrence, vancomycin was administered to treat the methicillin-resistant Staphylococcus. This case demonstrates the successful management and recovery of a bone marrow transplant patient with MDS who subsequently developed IE.

## Conclusions

We presented a case of a patient with MDS who had undergone BMT and was receiving steroid and immunosuppressive therapy, admitted due to acute heart failure. Detailed examinations revealed that IE had severely damaged the aortic valve cusps, resulting in acute aortic regurgitation. Emergency surgery, including aortic valve replacement and CABG, was successfully performed, ultimately saving the patient’s life. Given the rarity of reported cases of IE in post-bone marrow transplant patients, particularly those with MDS, we report this case to contribute to the literature on managing similar high-risk cases.
